# The Therapeutic Architecture of Chlorogenic Acids: Molecular Mechanisms in Chronic Disease Prevention

**DOI:** 10.3390/nu18152542

**Published:** 2026-08-04

**Authors:** Gabriela Morales-Lima, Mariana Esteves Felix Penha, Beatriz Silva Piristrello, Scarlett Cristina Mendes da Silva, Giuseppina Negri, Carlos A. Toro, Fúlvio Rieli Mendes, Giulio Maria Pasinetti

**Affiliations:** 1 Center for Natural and Human Sciences, Universidade Federal do ABC, São Bernardo do Campo 09606-045, SP, Brazil; gabriela.morales@ufabc.edu.br (G.M.-L.); mariana.felix@ufabc.edu.br (M.E.F.P.); beatriz.piristrello@aluno.ufabc.edu.br (B.S.P.); scarlett.cristina@aluno.ufabc.edu.br (S.C.M.d.S.); gnegri18@gmail.com (G.N.); fulvio.mendes@ufabc.edu.br (F.R.M.); 2 Spinal Cord Damage Research Center, James J. Peters VA Medical Center, Bronx, NY 10468, USA; carlos.torochacon@va.gov; 3 VA Sequencing Collaborations United for Research and Epidemiology (seqCURE), James J. Peters VA Medical Center, Bronx, NY 44106, USA; 4 The Bronx Veterans Medical Research Foundation, Bronx, NY 10468, USA; 5 Geriatrics Research, Education and Clinical Center (GRECC), James J. Peters VA Medical Center, Bronx, NY 10648, USA

**Keywords:** chlorogenic acids, polyphenols, coffee, *Ilex paraguariensis*, gut–brain axis, Nrf2 pathway

## Abstract

This review examines the structural variety, distribution, and physicochemical properties of chlorogenic acids (CGAs), identifying coffee and green coffee as the leading dietary sources of these compounds. Preclinical studies indicate that plant-derived phenolic compounds exhibit strong antioxidant, anti-inflammatory, neuroprotective, cardioprotective, and antidiabetic effects across various disease models. The biological effectiveness of CGAs is attributed to their modulation of cellular signaling pathways, particularly by activating the erythroid 2-related factor 2 antioxidant defense mechanism and inhibiting the pro-inflammatory nuclear factor kappa B pathway. The regulation of the energy-sensing Sirtuin 1 and AMP-activated protein kinase pathways further enhances these therapeutic effects. In the gastrointestinal tract, CGAs serve as key modulators of the microbiota–gut–brain axis by exerting prebiotic-like effects, lowering the *Firmicutes*/*Bacteroidetes* ratio, promoting the production of short-chain fatty acids, and preserving gut barrier integrity. Some preclinical studies with coffee, *Ilex paraguariensis*, *Eugenia uniflora*, and other CGAs-rich extracts are also discussed. However, despite strong preclinical evidence, translating these findings into human clinical settings remains inconsistent due to significant individual differences in gut microbiota metabolism and the confounding effects of other components in dietary supplements, such as caffeine. Considering this, the review will first explore the molecular mechanisms supporting the potential development of CGAs as preventive interventions, while also discussing current human trials demonstrating selective improvements in neurological, cardiovascular, and metabolic functions. It will also highlight a historical limitation: the lack of studies on the bioavailability, bioactivity, and efficacy of isolated CGAs. The review will conclude by addressing the constraints of clinical studies, emphasizing the urgent need for future precision nutrition frameworks that employ standardized CGAs formulations to enhance potential therapeutic outcomes.

## 1. Introduction

Chlorogenic acids (CGAs) constitute a family of phenolic compounds produced by plants, formed through the esterification between *trans*-cinnamic acid, such as *p*-coumaric, caffeic, ferulic, and sinapic acids, with quinic acid, including mainly caffeoylquinic acids, feruloylquinic acids, and *p*-coumaroylquinic acids. CGAs also include compounds formed with various quinic acid epimers [1,2,3].

The nomenclature of CGAs has historically been inconsistent, involving different conventions over time and, consequently, making direct comparison between studies difficult [1,2]. Given this, we adopt the following terminological convention: the term CGA (singular) is used to refer to any isolated compound from the chlorogenic acid family, except when clearly specified, while the term CGAs (plural) designates the chemical class as a whole, including compounds described above. Among the CGAs, the compounds 3-O-caffeoylquinic acid (3-CQA), known as neochlorogenic acid, and 5-O-caffeoylquinic acid (5-CQA), also called as chlorogenic acid, are the most extensively studied because of their higher occurrence in plant-derived foods and their biological properties [1,2,3]. It should be noted that this is a simplification adopted for readability purposes: the studies discussed in this review did not always use an isolated constituent and may involve plant extracts rich in CGAs or mixtures of isomers.

CGAs are widely distributed across several foods and plant species and are among the most abundant polyphenols in the human diet [4]. Their presence is frequently reported in common products, such as wine, olive oil, potatoes, coffee (*Coffea arabica*, *Coffea canephora = C. robusta*), green coffee, teas, including mulberry leaf (*Morus alba*) and mate (*Ilex paraguariensis*), fruits (such as strawberry, mango, and blueberries), vegetables, and medicinal plants traditionally used in folk medicine, including *Melissa officinalis*, *Eucommia ulmoides*, chrysanthemum (*Chrysanthemum morifolium*), and honeysuckle (*Lonicera* spp.) [1,5,6,7,8]. For a detailed description of dietary sources of CGAs, see Clifford et al. [1] and Lu et al. [9]. Among the main dietary sources of CGAs, coffee is the most prominent beverage due to its widespread consumption worldwide [9]. Notably, the origin and variety of coffee, its maturation state, the harvest process, and mainly the roasting degree influence the levels of CGAs [1,10,11,12].

Although the chlorogenic acid family comprises a wide range of structurally related compounds distributed in several plants, this review will focus primarily on CGAs present in dietary sources, given their preventive potential against chronic diseases and therapeutic importance for human nutrition. To achieve this, we discuss preclinical evidence utilizing both isolated compounds and CGAs-rich plant extracts, for a critical comparison between synergistic effects and direct, receptor-level pharmacological mechanisms. Finally, we intend to contextualize these preclinical findings with current clinical evidence to evaluate the translational viability and therapeutic relevance of CGAs in human nutrition and health.

### 1.1. Chemical Diversity and Physicochemical Aspects of Chlorogenic Acids

CGAs are a diverse group of polyphenols and include caffeoylquinic acids, feruloylquinic acids, and *p*-coumaroylquinic acids [13]. The terms “chlorogenic acids” and “acyl-quinic acids” have caused some confusion because many articles do not provide a clear definition of them. The lack of clarity in many articles is attributed to older nomenclature and the vast scope of this chemical family. Acyl-quinic acids are the most studied group among 400 chlorogenic acids [1,2].

The biosynthesis of CGAs occurs primarily via the shikimic acid pathway during aerobic respiration and depends on the following three key enzymes: phenylalanine ammonia-lyase, shikimic acid/quinic acid hydroxyl cinnamyl transferase, and quinic acid cinnamate hydroxyltransferase [5]. All biosynthesis steps involve the most important enzyme, hydroxycinnamoyl-CoA: quinate hydroxycinnamoyl transferase, because it can catalyze the conversion of caffeoyl-CoA and quinic acid into CGAs [3]. Differences in the number and position of caffeoyl groups attached to quinic acid give rise to different isomers, which are classified as monocaffeoylquinic and dicaffeoylquinic acids and their derivatives [5]. The main compounds include 1-, 3-, 4-, and 5-caffeoylquinic acids and 1,3-, 1,4-, 1,5-, 3,4-, 3,5-, and 4,5-dicaffeoylquinic acid derivatives [5]. The compounds 3-CQA and 5-CQA are structural isomers. The compound 5-CQA has the caffeoyl group attached to the carbon-5 position of the quinic acid ring, while 3-CQA has it at carbon-3. According to IUPAC numbering conventions, 5-CQA is designated as chlorogenic acid; however, other researchers refer to it as 3-CQA, its isomer with the caffeoyl group linked to the 3-position of 1L-(−)-quinic acid. The presence of many isomers is determined by the location and quantity of caffeoyl groups bonded to quinic acid. These isomers not only differ in structure but also exhibit unique pharmacological activities [3,14,15].

Besides the mono- and dicaffeoylquinic acids already mentioned, the CGAs family also comprises tri-esters and the single tetra-ester of caffeic acid with quinic acid (tricaffeoylquinic acids and tetracaffeoylquinic acids, respectively), as well as mixed di-esters formed between caffeic and ferulic acid, or between caffeic and sinapic acid. There are also mixed esters involving aliphatic dibasic acids, such as glutaric, oxalic, and succinic acids [14].

In addition to their structural diversity, CGAs have important physicochemical characteristics related to their absorption and metabolism. The compounds can be found as white or slightly yellow crystals, showing low solubility in organic solvents such as chloroform, ether, and benzene, and high solubility in polar solvents, including methanol, ethanol, and acetone [5]. Isolating CGAs from plant material has historically relied on conventional solvent-based approaches, including maceration, reflux, and Soxhlet extraction, with the latter typically yielding the greatest recovery of these compounds [16]. Despite their widespread use, these methods have practical drawbacks: they tend to require long processing times, consume considerable amounts of solvent, and often yield results that are difficult to reproduce consistently [14,17]. It is worth noting that the physicochemical properties of CGAs depend on the identity, number, and position of the acyl residues esterified with the quinic acid as well as on the functional groups present on the aromatic moiety of the acyl residues [3]. Besides this, the process of acyl migration, which can be intra- and/or intermolecular, is temperature- and pH-dependent, with the increased transesterification taking place at basic pH. This transesterification reaction is responsible for regioisomerization of appropriate CQAs and occurs through migration of cinnamoyl moieties from one quinic acid alcohol group to another [14].

The stability of CGAs can be influenced by their sensitivity to heat, pH, and light, caused by their ester bonds and phenolic hydroxyl groups. High temperatures can trigger hydrolysis, isomerization, and oxidation reactions [15]. Coffee is considered the main dietary source of CGAs, and green coffee beans stand out because the roasting process can significantly alter the composition of CGAs. During roasting, part of the CGAs undergo the chemical process of intramolecular cyclization and are converted into lactones (also called quinides), through the loss of a water molecule and the formation of an intramolecular ester bond [18]. Importantly, some of the newly formed constituents, like quinides (derived from CGAs) and melanoidins (high-molecular-weight nitrogenous polymers derived from sugars and proteins), exhibit important biological activities including antioxidant, anti-inflammatory, and antimicrobial properties [19,20,21].

Król et al. [10] quantified total CGAs content in roasted *Coffea arabica* beans and found that both origin and roasting conditions significantly influenced final polyphenol levels. In a study tracking seven individual CGAs across the roasting process in both Arabica and Robusta beans, Trugo and Macrae [22] documented a roast-dependent decline of CGAs content: mild roasting conditions eliminated approximately 60% of total CGAs, while severe roasting left almost none intact. Notably, it was observed that distinct CGA isomers may degrade differentially even under the same roast conditions. For example, light roast led to less than 7% loss of 3-CQA content, whereas the same condition caused a loss around 72% of 5-CQA both in Arabica and Robusta beans [22]. Using both water and ethanolic extracts prepared from green and roasted beans, Budryn et al. [23] observed that harsher extraction protocols, particularly boiling coffee under normal or elevated pressure, drove a progressive conversion of 5-CQA into its 3- and 4-CQA counterparts in both coffee species. Comparing dark-roasted samples to their green counterparts, total CGA content was 14-fold lower in Robusta versus only about 5-fold in Arabica. Additionally, Kim et al. [12] investigated how roasting degree affects the physicochemical behavior of CGA in green versus roasted coffee bean extracts. Using liquid chromatography–mass spectrometry, they identified four distinct CGA isomers in green coffee and showed that CGAs content decreased progressively with roasting intensity, while its degradation products, caffeic and quinic acid, accumulated and altered the extract’s pH. Confirming other studies, the authors showed that some isomers identified did not degrade at the same rate, reinforcing the idea that roasting does not affect all CGAs isomers uniformly.

### 1.2. Pharmacokinetics of CGAs

The compound 5-CQA undergoes rapid degradation through oxidation, isomerization, and enzymatic hydrolysis during storage and under gastrointestinal conditions, and its intestinal absorption is limited due to its low physicochemical stability and low aqueous solubility [24]. CGAs that remain intact reach the small intestine but are poorly absorbed there, while the corresponding cinnamic acids are efficiently absorbed after hydrolysis by digestive or microbial enzymes in the colon [1]. After oral ingestion, approximately one-third of CGAs are rapidly absorbed in the upper gastrointestinal tract [25], while the remaining fraction can be metabolized by enzymes produced by the intestinal microbiota, especially by bacteria such as *Lactobacillus gasseri*, *Bifidobacterium lactis*, and *Escherichia coli*, which release quinic acid and chlorogenic acid for subsequent intestinal absorption [13].

In fact, Stalmach et al. [26] monitored CGA levels in the plasma and urine of 11 volunteers after consuming instant coffee containing 412 µmol of CGAs. This study reported that the detected CGAs reached plasma peak concentrations (Cmax) ranging from about 2 to 16 nM within one hour (Tmax), suggesting small intestine absorption. Notably, the metabolite profile indicates the presence of glucuronide and sulphate conjugates, and higher values of Cmax and Tmax were detected, suggesting colonic absorption and bacterial catabolism. Indeed, urine analysis evidenced that CGAs and their metabolites are well absorbed, with an elimination of only around 29% of the consumed CGAs [26]; therefore, the absorption was impaired in patients who underwent ileostomy [25]. Accordingly, Monteiro et al. [27] reported a Cmax ranging from 1 to 5 µmol/L and peak concentration being reached within 2.3 h after coffee consumption. The authors also highlight a large variability across subjects, probably due to cytochrome P450 and other carrier system polymorphisms [27].

Regarding penetration into the central nervous system, a study with patients who underwent lumbar puncture identified caffeic acid, one of the main metabolites derived from CGAs, in cerebrospinal fluid samples, with plasma caffeic acid concentrations that did not correlate with those found in the cerebrospinal fluid, suggesting that transport across the blood–brain barrier does not occur by simple or facilitated diffusion [28]. Instead, this study suggests that caffeic acid transportation may occur through monocarboxylic acid transporters, present both in intestinal cells and in the capillary endothelial cells of the blood–brain barrier. Furthermore, it is worth noting that caffeic acid is subjected to the conjugation process (e.g., methylation, sulphatation and glucuronidation), which is known to impact blood–brain barrier permeability [28].

On the other hand, Bai et al. [29] monitored plasma and cerebrospinal fluid levels of 5-CQA in rats 5 min after acute tail vein injection. It was reported that 5-CQA can cross the blood–brain barrier, although with a limited permeability (below 0.5%). This study also demonstrated that 5-CQA half-life is shorter in the cerebrospinal fluid than in the plasma, and that 5-CQA concentration varies across different brain regions and time points after administration [29]. Taken together, the studies indicate that CGAs are both absorbed unaltered and subjected to metabolization by gut microbiota, following hepatic and other processes of biotransformation; therefore, only a small part of the CGAs and most likely their metabolites reach the CNS and other target organs. For more information regarding pharmacokinetics of CGAs, see Clifford et al. [1] and Lu et al. [9].

## 2. Methodology

For this narrative review, a comprehensive search of the literature was conducted across databases PubMed and Google Scholar. The primary search focused on the CGAs and their specific pathways, combining keywords regarding the chemical structure (“chlorogenic acid”, “chlorogenic acids”, and “caffeoylquinic acid”) with relevant molecular mechanisms (“Nrf2/KEAP1 signaling”, “NF-*κ*B”, “AMPK”, “ROS”, “MAPK pathway”, “oxidative stress”, “inflammation”, and “gut–brain axis”) and clinical or pathological contexts (“neuroprotection”, “cardiovascular” and “anticancer”). Following the primary identification of the key natural matrices rich in chlorogenic acids most discussed in the literature, a secondary search was executed targeting CGAs-rich plant species to evaluate how their complex extracts or traditional uses align with CGAs-mediated bioactivity. The inclusion criteria encompassed peer-reviewed articles, high-quality mechanistic studies, preclinical research (both in vitro and in vivo), and clinical trials published in English.

## 3. Main Pharmacological Effects Described for Chlorogenic Acids

### 3.1. Antioxidant Effect

The production of reactive oxygen species (ROS) is a natural process and is essential for maintaining homeostasis. However, when ROS production becomes excessive, homeostasis is disrupted, overwhelming the antioxidant system and leading to cellular stress and damage. Continuous exposure to excess reactive species can result in tissue damage and a wide range of diseases [30]. CGAs, like other polyphenols, are widely recognized for their antioxidant effects. This capability stems from their molecular structure, in which the presence of hydroxyl groups enables them to donate hydrogen atoms or electrons to unstable free radicals in the environment, thereby reducing or even halting the damage they cause [16,31].

Numerous studies have explored the antioxidant potential of CGAs and their possible applications. Several studies have reported a reduction in ROS production accompanied by increased activity of endogenous antioxidant enzymes, including superoxide dismutase (SOD), catalase (CAT), and glutathione peroxidase (GPx), indicating that CGAs may reinforce cellular antioxidant defenses rather than acting solely as a chemical antioxidant [32,33]. In addition, CGAs suppress nitric oxide production in activated macrophages and reduce NADPH oxidase activity, both of which are important sources of oxidative and inflammatory stress [34,35].

Because oxidative stress can affect various systems in the body, its implications have been actively explored across several disease models. Improvements in antioxidant status have been observed in intestinal immune stress models, in which CGA supplementation enhanced the antioxidant capacity and reduced the production of inflammatory mediators [36]. Similar findings have been reported in oligodendrocyte-like cells, where CGA reduced ROS accumulation and apoptosis while simultaneously promoting cellular differentiation, suggesting a possible role in tissue repair and remyelination [37].

Additionally, Neelakandan et al. [38] evaluated the effects of CGA-loaded nanoparticles on oxidative stress in a skin cancer model using several biomarkers, including glutathione (GSH), glutathione reductase, glutathione S-transferase, SOD, CAT, and GPx. The levels of thiobarbituric acid reactive substances, an indicator of lipid peroxidation, were also evaluated in plasma and skin tissues, providing a comprehensive assessment [38]. In a hypoxia-reoxygenation model using human hepatocytes from the HL7702 cell line and cells overexpressing TLR-4, pretreatment with CGAs reversed the increase in inflammatory and pro-apoptotic proteins, inhibited ROS production during reoxygenation, and reduced mitochondrial damage [39]. These findings demonstrate that CGAs provide antioxidant protection through several mechanisms, supporting their potential use in conditions involving oxidative damage.

### 3.2. Anti-Inflammatory Effect

Inflammation is a fundamental biological response activated following tissue injury, infection, or exposure to harmful stimuli. Under normal circumstances, inflammatory processes facilitate pathogen elimination and repair. However, persistent or excessive activation of inflammatory pathways can result in chronic tissue damage and contribute to the pathogenesis of numerous diseases, including metabolic, cardiovascular, and neurodegenerative disorders [40].

The anti-inflammatory activity of CGAs is closely linked to their ability to modulate oxidative stress and intracellular signaling pathways. As ROS are important mediators of inflammatory responses, the antioxidant properties of CGAs may indirectly contribute to the suppression of inflammation. Several studies have reported a decrease in ROS production and pro-inflammatory cytokines, such as tumor necrosis factor alpha (TNF-α), interleukin 1 beta (IL-1β), and interleukin 6 (IL-6), following CGA treatment [37,41,42].

At the molecular level, CGAs regulate several key signaling pathways involved in inflammatory responses, such as the nuclear factor kappa B (NF-*κ*B), AMP-activated protein kinase (AMPK), mitogen-activated protein kinase (MAPK), and toll-like receptor 4 (TLR4) mediated pathways. For instance, evidence from renal fibrosis and bovine mammary epithelial cell models demonstrated that the compound CGA suppresses NF-*κ*B activation, resulting in reduced expression of inflammatory mediators [43,44]. Similar anti-inflammatory effects were reported by Zhang et al. [45] in a rat model of pneumonia, where inhibition of the p38-MAPK signaling pathway was associated with reduced inflammatory injury and attenuation of disease progression.

The anti-inflammatory properties of CGAs are associated with improvements in metabolic disorders. In diabetic cardiomyopathy, CGA and ferulic acid reduced cardiac lipotoxicity, apoptosis, and mitochondrial dysfunction in models of diabetic cardiomyopathy, acting through the GCGR/PPARα and GCGR/AMPK pathways [46]. Taken together, these findings suggest that CGAs act on multiple targets involved in inflammatory signaling and may be a promising candidate for treating conditions characterized by chronic inflammation.

### 3.3. Modulation of the Nrf2 Pathway

ROS are physiological byproducts of cellular metabolism; however, excessive ROS release leads to a redox imbalance and oxidative stress, which are key elements in several pathological conditions, including neurological, metabolic, and cardiovascular diseases. The nuclear factor erythroid 2-related factor 2 (Nrf2) pathway is an important defense mechanism against oxidative stress and is considered the master regulator of the antioxidant response [47,48,49].

Briefly, Nrf2 is a transcription factor that remains in the cytosol under homeostatic conditions, coupled with Kelch-like ECH-associated protein 1 (KEAP1), which acts as an Nrf2 repressor by favoring its ubiquitination and degradation through the proteasome 26S complex. However, under oxidative stress, KEAP1 dissociates from Nrf2, promoting its nuclear translocation, where Nrf2 binds to the antioxidant response element (ARE). Thus, the synthesis of antioxidant enzymes is induced, e.g., SOD, CAT, GPx, and heme oxygenase-1 (HO-1), among others [47,48,49]. Furthermore, the Nrf2 pathway intrinsically interacts with the NF-*κ*B inflammatory response pathway, acting as an inhibitor of its nuclear translocation and indirectly modulating cytokine release, cell adhesion, and proteostasis [50]. Moreover, the Nrf2-NF-*κ*B interplay has been shown to be associated with a cognitive-enhancing effect [51].

Considering the mechanisms outlined above, Nrf2 inducers can be a potential therapeutic approach for various diseases by reducing ROS levels, improving mitochondrial function, and attenuating the inflammatory response [52,53]. Several plant secondary metabolites have been shown to activate the Nrf2 pathway [54]. In fact, a molecular docking study demonstrated that CGA acts as an Nrf2 inducer by competing with KEAP1 for binding, thereby favoring Nrf2 release and translocation to the nucleus [55]. Moreover, Liang et al. [56] showed that distinct CGA isomers confer different antioxidant capacity in terms of radical scavenging and regulation of Nrf2 pathway-related genes in Caco-2 cells. The same study hypothesized that CGAs’ radical-scavenging effect is a key step in producing phenoxyl radicals, which, in turn, allow direct molecular contact with Keap1 cysteine residues, favoring Keap1-Nrf2 oxidation and triggering Nrf2 nuclear translocation and its consequent downstream modulation. Indeed, this hypothesis was supported by molecular dynamics simulations. Taken together, these results demonstrate that CGAs’ antioxidant capacity may be attributed to the combination of their free-radical-scavenging potential and the modulation of the intracellular redox status [56].

Interestingly, Tang et al. [57] showed that CGA ameliorated anxiety and cognitive impairment in a post-traumatic stress disorder rat model of single prolonged stress by the regulation of Nrf2-NF-*κ*B pathway interplay. Zheng et al. [58] investigated the neuroprotective effects of CGA against ischemic–hypoxic brain injury. The results demonstrated that treatment with CGA significantly reduced cerebral infarct volume and edema and improved cognitive function and long-term spatial memory, as assessed by the Morris water maze test. There was a decrease in malondialdehyde (MDA), TNF-α, and IL-1β levels, whereas CAT activity and SOD2 levels increased. The suggested mechanism of action involves the activation of the sirtuin 1 (SIRT1) axis, which in turn regulates the Nrf2-NF-*κ*B pathway, resulting in reduced neuronal apoptosis and suppression of excessive astrocyte activation [58].

Accordingly, Sun et al. [59] focused on epilepsy-related models in which oxidative stress and inflammatory responses contribute significantly to neuronal injury. These authors demonstrated that the administration of CGA reduced seizure severity, improved cognitive performance, and decreased neuronal loss within the hippocampus of mice with pentylenetetrazole-induced epilepsy. They linked these effects to the regulation of microglial polarization and to the activation of the Nrf2/HO-1 antioxidant pathway [59]. Thus, oxidative stress plays a pivotal role in gut–brain axis homeostasis, with some evidence suggesting that Nrf2 is relevant to this communication [60,61,62].

### 3.4. Effects on the Microbiota–Gut–Brain Axis

The crosstalk among the central, autonomic, and enteric nervous systems comprises the gut–brain axis, with several mechanisms, including immune and endocrine pathways, modulating this multifaceted communication [63,64]. Moreover, the gut microbiome is a critical modulator of this system, and dysbiosis is frequently correlated with several neurological and psychiatric disorders [65,66,67]. In this context, the reestablishment of gut microbiota homeostasis is highlighted as a promising therapeutic approach, with several dietary polyphenols shown to modulate gut–brain communication [68,69,70]. CGAs are mostly absorbed in the colon, where they interact with the resident microbiota and are metabolized into active metabolites, thereby making CGAs compounds with a prebiotic effect that may be beneficial for gut–brain axis-related diseases [25,71].

Importantly, it is not unusual for patients with inflammatory bowel disease to be diagnosed with psychiatric disorders [72], and alterations in bowel habits are frequent complaints among patients with mood disorders [73]. Studies have shown that CGA and CGA-rich extracts can counteract cognitive dysfunction and anxiety- and depression-like behaviors in colitis mouse models [62,74], as well as in chronic unpredictable stress [61] and adrenocorticotropic hormone-induced depression models [75]. Remarkably, these cognitive-enhancing effects have consistently been associated with the restoration of gut microbiome homeostasis through the modulation of inflammatory cytokine levels, oxidative stress, and gut barrier stability [61,62,74,75].

There is interesting evidence suggesting a potential role of CGAs in the Western diet (also known as the Standard American Diet), a modern eating pattern characterized by high intakes of pre-packaged foods, refined grains, added sugars, red and processed meats, fried foods, and high-fat dairy, which is frequently associated with the development of metabolic syndrome and its outcomes, including systemic inflammation, dysbiosis, and cognitive impairment [76]. A study showed that dietary CGAs improved high-fat, high-fructose diet-induced changes in metabolic and synaptic functions, as well as cognitive impairment in mice, with this effect closely related to gut microbiome restoration [77]. Additionally, an apple polyphenol extract rich in CGAs has been shown to improve high-fructose diet-induced cognitive impairment and depressive- and anxiety-like behaviors in mice while alleviating histopathological damage in the liver, colon, and brain tissues [78]. The same study showed that this extract modulated the hypothalamic–pituitary–adrenal axis, bile acid circulation, and gut microbiome diversity. Metabolic syndrome is also linked to the development of non-alcoholic fatty liver disease, and it was previously shown that CGAs ameliorate its effects by regulating the gut microbiota and, consequently, modulating glucagon-like peptide-1 in the portal vein of high-fat-fed mice [79].

It has been reported that the gut microbiome is also implicated in sleep disorders [80], and CGAs have been shown to attenuate cognitive impairment and anxiety in sleep-deprived mice. In addition, CGA exhibits anti-inflammatory and antioxidant effects, protects the intestinal barrier, and modulates the gut microbiota by increasing the proportion of beneficial bacteria and decreasing that of opportunistic bacteria [60]. Nevertheless, it is worth noting that the individual microbiota modulates polyphenols’ bioavailability and consequently their cognitive-enhancer effects. A study showed that cognitive resilience promoted by a polyphenol-rich diet in mice that underwent sleep deprivation was impaired by antibiotic-induced dysbiosis [81].

Lin et al. [82] studied intestinal mucositis induced by 5-fluorouracil in mice treated with CGA. The results showed that it reduced MDA levels, increased glutathione levels, restored the expression of antioxidant enzymes (CAT, SOD1, and GPx3), and reduced serum levels of nitric oxide and the cytokines TNF-α, IL-6, and IL-1β. It activated the AMPK/SIRT1 axis, inhibited TLR4/NF-*κ*B pathway activation, and reduced weight loss, intestinal damage, and oral mucosal lesions. It also reduced the levels of Bax and cleaved caspase-3 proteins, increased the levels of the anti-apoptotic protein Bcl-2, and reduced the levels of the autophagy markers LC3-II/I, Beclin 1, and p62 [82]. Lastly, CGAs showed protective effects against toxicity induced by environmental pollutants, including lead and trimethyltin chloride, ameliorating cognitive function while modulating inflammatory signaling pathways and the gut microbiota [83,84].

Furthermore, short-chain fatty acids (SCFAs; e.g., acetate, propionate, and butyrate) are microbiome fermentation byproducts strongly influenced by diet and implicated in various diseases, with modulatory effects on immune response, barrier integrity, and metabolic function [85,86]. Although SCFAs are mainly produced in the colon, they can reach the CNS via the bloodstream because they can cross the BBB, influencing microglial function. Moreover, lower levels of SCFAs have been associated with an increased risk of inflammatory diseases [86]. In this context, CGAs have been shown to positively modulate the abundance of SCFA-producing bacterial genera, as well as improve SCFAs levels [74,75,77,83,84].

Although the disease models that assess the role of CGAs in the gut–brain axis are diverse, they share key elements and markers (Table 1), including modulation of the *Firmicutes*/*Bacteroidetes* (F/B) ratio in the gut microbiome and the production of SCFAs. Thus, synaptic function and neurotransmission, even as inflammation and oxidative response signaling, including NF-*κ*B and Nrf2 pathways, are frequently cited as mediators of the protective effects in these studies [60,61,62,74,77,83,84]. Notably, these studies mostly rely on concomitant changes affecting peripheral and central outcomes, sustaining a correlation association but still lacking evidence for a causal relationship.

### 3.5. Antidiabetic Effect

The antidiabetic effects of CGAs have been investigated in several experimental models, revealing actions that extend beyond glucose regulation. Current evidence suggests that CGAs influence multiple aspects of metabolic homeostasis, including insulin sensitivity, lipid metabolism, oxidative stress, inflammation, and pancreatic β-cell function [87,88,89].

Using the db/db mouse model, which carries a spontaneous homozygous mutation in the leptin receptor gene, a gold-standard genetically modified animal model of type 2 diabetes mellitus and morbid obesity, CGA supplementation improved glucose tolerance and insulin sensitivity while reducing body weight gain and hepatic lipid accumulation [87]. These metabolic improvements were associated with increased expression of genes involved in fatty acid oxidation and lipolysis and reduced expression of genes linked to lipid storage. Restoration of antioxidant enzyme expression and modulation of inflammatory cytokines have also been observed, indicating that improvements in metabolic control may result from the coordinated effects on several biological pathways [87]. Similar benefits have been reported in prediabetic mice, where CGA improved insulin resistance and hepatic lipid metabolism. Interestingly, some of these effects are comparable to those achieved through regular physical exercise, emphasizing the broad metabolic influence of this compound [88].

In addition to their effects on peripheral tissues, CGAs may help preserve pancreatic β-cell function. Ihara et al. [89] demonstrated that CGA counteracts tunicamycin-induced endoplasmic reticulum stress by increasing cAMP response element-binding protein (CREB) phosphorylation and insulin receptor substrate-2 expression, two factors involved in β-cell survival and insulin responsiveness. The preservation of β-cell integrity is particularly relevant because progressive β-cell dysfunction is a hallmark of type 2 diabetes [89]. Evidence also suggests that CGAs may mitigate diabetes-associated complications, as evaluated by Saputri et al. [90] using a model of diabetic rats, where treatment with CGA improved cognitive performance while reducing oxidative stress and apoptosis in the frontal cortex. These changes were accompanied by increased expression of antioxidant enzymes and anti-apoptotic proteins, supporting the hypothesis that CGAs may protect neural tissue from the adverse effects of chronic hyperglycemia [90]. Collectively, these findings indicate that CGAs may contribute to glycemic control and protection against secondary complications associated with diabetes.

In addition to glycemic modulation, CGAs appear to exert beneficial effects on lipid metabolism. In a model of lipid metabolic dysfunction, Wang et al. [91] demonstrated that C2C12 cells treated with CGA exhibited improved mitochondrial function and reduced accumulation of free fatty acids and lactate through action on the LDHA–lactate axis. Furthermore, in mice fed a high-fat diet, CGA promoted a reduction in serum total cholesterol, triglycerides, and free fatty acids; promoted the transition to more oxidative muscle fibers, increasing mitochondrial biogenesis and function; increased endurance time in a treadmill exhaustion test; and led to changes in MDA reduction and increased SOD and CAT activity [91]. Further evidence supports the potential of CGA to counteract obesity-associated metabolic dysfunction, as its administration improved glucose and lipid metabolism while reducing oxidative stress, inflammation, and dysregulated appetite control in a high-fat diet-induced model [92]. However, the metabolic effects of CGA may also be influenced by the gut microbiota, as obesity-associated changes in microbial composition and activity can modify its biotransformation and potentially affect the production of bioactive metabolites [93]. Together, these findings highlight the multifaceted metabolic actions of CGA and suggest that differences in its metabolism and bioavailability may contribute to variability in its antidiabetic effects.

### 3.6. Cardiovascular Effect

Cardiovascular diseases are strongly influenced by oxidative stress, inflammation, fibrosis, and metabolic dysregulation, all of which have been identified as targets of CGAs. Consequently, numerous studies have explored the cardioprotective potential of these polyphenols in different experimental settings.

One of the most extensively investigated areas is myocardial injury. In myocardial ischemia–reperfusion models, CGA reduced inflammatory responses and pyroptotic cell death by inhibiting the long non-coding Neat1/NLRP3 inflammasome pathway, resulting in significant protection of cardiac tissue [94]. Similarly, in pressure overload-induced heart failure, treatment with CGA improved cardiac function and attenuated myocardial fibrosis. These effects are associated with the inhibition of STING—stimulator of interferon genes—protein signaling, suppression of NF-*κ*B activation, and reduced recruitment of inflammatory CCR2+ macrophages [95].

The cardiovascular benefits of CGAs extend beyond direct cardiac protection effects. Previous studies have demonstrated their antihypertensive effects, including reductions in blood pressure and inhibition of enzymes involved in vascular dysfunction, such as angiotensin-converting enzyme, cholinesterase, and arginase [96]. These observations suggest that CGAs influence both vascular tone and endothelial function. Furthermore, CGAs appear to modulate lipid metabolism, which is a key determinant of cardiovascular risk. Experimental evidence indicates reductions in body weight, serum cholesterol levels, and hepatic lipid accumulation. Mechanistically, these effects have been linked to the regulation of the AMPK, PXR, and SREBP2 signaling pathways, leading to decreased expression of proteins involved in cholesterol absorption and synthesis, including NPC1L1 and HMGCR [97].

Overall, the available evidence suggests that CGAs exert cardiovascular protection through a combination of antioxidant, anti-inflammatory, antifibrotic, antihypertensive, and lipid-regulating effects. Although most studies remain preclinical, the consistency of these findings across different models highlights the therapeutic potential of CGAs in the management of cardiovascular disorders.

### 3.7. Anticancer Effect

The potential anticancer effect of CGAs has been evaluated in tumoral cell lines and in vivo models. Vélez-Vargas et al. [98] evaluated the anticancer properties of chlorogenic acid in colorectal cancer. The compound significantly reduced the viability of SW480 cells, but no significant effect was observed on HT-29 cells. However, the CGA successfully induced an increase in ROS production and DNA fragmentation in both cell lines, further supporting its antiproliferative and pro-apoptotic potentials. In another study, even at lower concentrations, CGA reduced the viability of HT29 cancer cells, along with cell cycle arrest at the G0/G1 phase and induction of apoptosis at a concentration of 100 µM, through increased levels of caspases 3 and 9, increased levels of the pro-apoptotic protein Bax, and decreased levels of the anti-apoptotic protein Bcl-2 and NF-*κ*B [99]. Interestingly, a combination of CGA and caffeine also exhibited chemopreventive effects in vivo, significantly reducing aberrant crypt foci formation, decreasing cellular proliferation (Ki-67), increasing cleaved caspase-3, suppressing the oncomiR miR-21a-5p, and lowering IL-6, IL-17, and TNF-α levels, suggesting that this combination may contribute to colorectal cancer prevention by modulating inflammatory and epigenetic pathways [100].

In breast cancer cell lines, CGA inhibited viability and proliferation, induced apoptosis, and suppressed migration capacity. These findings were corroborated in vivo; in a subcutaneous tumor model in female mice, in which CGA reduced tumor volume and weight, CGA increased the proportion of CD4+ and CD8+ T cells, improved antitumor immunity, and prolonged survival [101]. In addition, a study showed that CGA reduced tumor weight and volume in 4T1 breast cancer BALB/c mice, as well as reduced metastatic nodules, an effect associated with the regulation of apoptosis pathways mediated by Bax, Bcl-2, caspase-3, and p53, with these effects being observed both in a preventive treatment protocol and as a treatment after tumor incidence [102]. These findings are further supported by evidence showing that CGA suppresses epithelial–mesenchymal transition, an important feature in breast cancer metastasis, through inhibition of the Wnt/β-catenin signaling pathway via downregulation of LRP6, increasing epithelial markers while reducing mesenchymal markers, as well as the activity of the matrix metalloproteinases 2 and 9, ultimately limiting tumor migration and invasion [103].

Despite CGAs’ anticancer potential in vitro, Matsuda et al. [104] reported their potential in attenuating the neurotoxicity induced by the antineoplastic drug bortezomib in PC12 and SH-SY5Y cells. Moreover, CGA restored the SubG1 cell population distribution and inhibited apoptosis in bortezomib-treated PC12 cells. However, this neuroprotection is accompanied by an inhibition of bortezomib anticancer activity in human squamous carcinoma cell lines, an effect hypothesized to be related to the conjugation of the CGA catechol group with the boronic acid group from bortezomib [104].

Furthermore, it has been suggested that CGAs’ antioxidant properties may also help prevent tumor development, as shown by Neelakandan et al. [38] in a mouse skin cancer model, where CGA-loaded nanoparticles restored antioxidant defenses and significantly reduced tumor formation, especially when applied topically. In addition to its direct effects on tumor cells, emerging evidence indicates that CGAs may also modulate the tumor microenvironment. In colorectal cancer, CGA reduced the production of the gut microbiota-derived metabolite 4-hydroxyphenylacetic acid, decreasing the recruitment of immunosuppressive polymorphonuclear myeloid-derived suppressor cells (PMN-MDSCs) through inhibition of the JAK2/STAT3/CXCL3 signaling axis, partially restoring antitumor immune responses and suggesting a potential role in improving immunotherapy outcomes [105]. Similarly, CGA suppressed IFN-γ-induced PD-L1 expression by inhibiting the JAK/STAT1/IRF1 signaling pathway, thereby enhancing T-cell-mediated antitumor immunity and significantly improving the efficacy of anti-PD-1 therapy in murine tumor models, suggesting that CGA may improve the efficacy of cancer immunotherapy [106].

Although preclinical studies consistently demonstrate the anticancer potential of CGAs, additional investigations are needed to clarify the relationship between their bioavailability, bioactivity, and therapeutic efficacy. In this context, recent advances in nanoformulation have shown promising results by enhancing the antitumor activity of CGAs in colorectal cancer models, suggesting that optimized delivery systems may help overcome some of the current translational challenges [105].

### 3.8. Neuroprotective Effect

Increasing evidence suggests that CGAs exert protective effects in the central nervous system through mechanisms involving the regulation of oxidative stress, neuroinflammation, apoptosis, and neuronal plasticity [13,107]. Because these processes are common features of many neurological disorders, the neuroprotective potential of CGAs has attracted considerable research interest in recent years.

Pretreatment with CGA reduced oxidative stress, neuroinflammation, cerebral infarction, and neuronal damage in rat models of neonatal hypoxic–ischemic brain injury. It also improved cognitive and learning functions by activating the Sirt1/Nrf2/NF-*κ*B pathway [58]. CGA also demonstrated a significant neuroprotective effect in status epilepticus models, reducing oxidative stress and lipid peroxidation, mechanisms directly linked to seizure-induced neuronal degeneration [108]. In a model of cellular senescence induced by hydrogen peroxide exposure, CGA restored Lamina B1 levels and reduced the activation of the p53 and p21 pathways, as well as MAPK and NF-*κ*B signaling. CGA also restored insulin signaling through the PI3K-AKT-GLUT4 axis. Their findings suggest that CGA attenuates senescence markers, modulates cellular metabolism, and reduces oxidative stress [109].

Experimental studies investigating amyloid-β (Aβ) toxicity and pharmacologically induced cognitive impairment have demonstrated improvements in memory performance, synaptic integrity, and neuronal plasticity following CGAs treatment. These beneficial effects were accompanied by a reduction in Aβ accumulation and neuroinflammatory markers [110,111]. Additional evidence indicates that CGA promotes dendritic remodeling and decreases lipid peroxidation in neural tissue, factors that may contribute to the preservation of cognitive function and neuronal resilience during aging or disease progression [112]. In the neuroblastoma SH-SY5Y cell line, 1,3-di-caffeoylquinic acid demonstrated a high affinity for Aβ (1–40) and inhibited its aggregation. It restored cell viability by reducing ROS levels, stabilizing the mitochondrial membrane potential, and inhibiting apoptosis [113]. In a model of cognitive impairment, CGA restored cAMP levels, upregulated CREB and brain-derived neurotrophic factor (BDNF) gene expression, attenuated memory and locomotor deficits, and reduced neuronal damage and Aβ accumulation in scopolamine-treated mice [111].

In a cell model of PD, pretreatment with CGA protected SH-SY5Y cells and primary cortical neurons from 6-hydroxydopamine toxicity. CGA protects cells from reduced neuronal viability by preventing the inactivation of the Akt/mTOR pathway and inhibiting excessive activation of the Erk1/2 pathway [114]. A dose-dependent cytoprotective effect of CGA was also observed in an in vitro study of α-synuclein-related toxicity in PC12 cells [115]. In a mouse model of striatal neurodegeneration induced by 3-nitropropionic acid, intraperitoneal administration of CGA produced an anti-inflammatory effect by maintaining TNF-α, IL-6, and IL-2 levels close to those of the control group, increasing IL-4 levels in the striatum and frontal cortex, and preventing lipid peroxidation [116]. Additionally, in a Parkinson’s disease model using A53T transgenic mice, intragastric treatment with CGA improved gait coordination and step frequency and restored grip strength. At the molecular level, the treatment prevented the reduction in tyrosine hydroxylase expression, suppressed p-Erk1/2 activation in the cortex and striatum, and prevented the decrease in p-Akt and p-mTOR, thereby reducing neuronal apoptosis in the brain [114].

**Figure 1:**
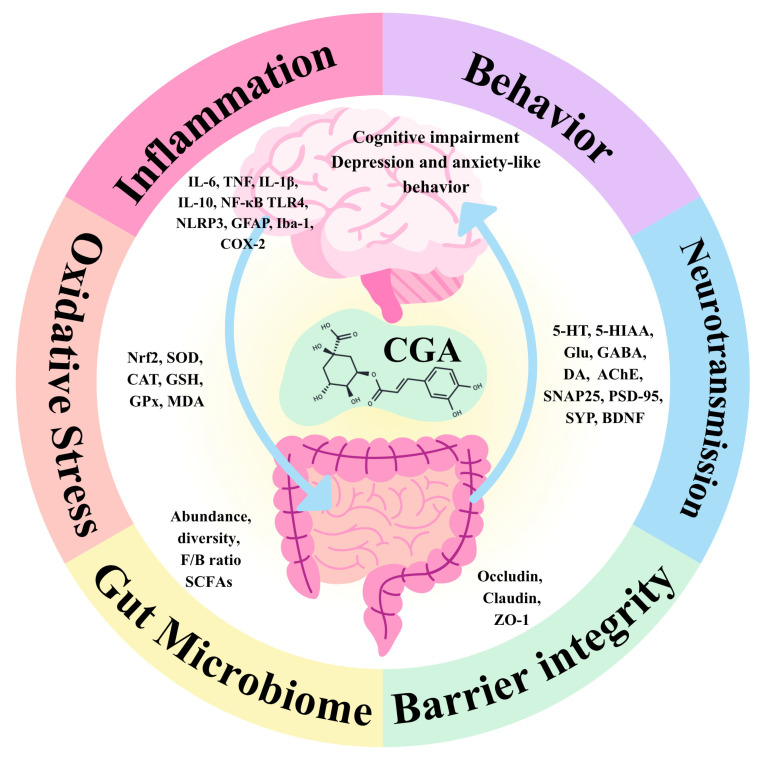
Main pathways involved in the beneficial effects of CGA and CGA-rich extracts. 5-HIAA: 5-hydroxyindoleacetic acid; 5-HT: serotonin; AChE: acetylcholinesterase; BDNF: brain-derived neurotrophic factor; CAT: catalase; COX-2: cyclooxygenase-2; DA: dopamine; F/B ratio: *Firmicutes*/*Bacteroidetes* ratio; GABA: gamma-aminobutyric acid; GFAP: glial fibrillary acidic protein; Glu: glutamate; GPx: glutathione peroxidase; GSH: glutathione; Iba-1: ionized calcium-binding adaptor molecule 1; IL-1β: interleukin 1β; IL-10: interleukin 10; IL-6: interleukin 6; MDA: malondialdehyde; NF-*κ*B: nuclear factor kappa B; NLRP3: NLR family pyrin domain containing 3; Nrf2: nuclear factor erythroid 2-related factor 2; PSD-95: postsynaptic density protein 95; SCFAs: short-chain fatty acids; SNAP25: synaptosomal-associated protein 25; SOD: superoxide dismutase; SYP: synaptophysin; TLR4: toll-like receptor 4; TNF: tumor necrosis factor; ZO-1: zonula occludens-1.

Taken together, the preclinical results support the therapeutic potential of CGAs in age-related chronic and neurodegenerative diseases. The main pathways involved in the beneficial effects of CGAs are summarized in Figure 1. For more details, see Lu et al. [9], Nguyen et al. [13] and Zeng et al. [107].

## 4. Studies with Plant Extracts Rich in CGAs

Plants rich in CGAs exhibit potent antioxidant and anti-inflammatory activities, which may contribute to their potential neuroprotective effects (Table 2). Several coffee components have shown neuroprotective effects in in vitro assays and experimental models of Parkinson’s disease [115,117,118]. A green coffee extract containing 21% chlorogenic acid and 27–33% caffeoylquinic acid was evaluated in animal models of Parkinson’s disease. The treatment reduced haloperidol-induced catalepsy in mice and had limited effects on the unilateral lesion of the medial forebrain bundle induced by 6-hydroxidopamine in rats [119]. The pro-cognitive effect of a green coffee extract containing CGAs and eicosanoyl-5-hydroxytryptamide was also examined in mice with scopolamine-induced amnesia. The extract improved the animal’s performance while ex vivo analysis revealed that it inhibited brain acetylcholinesterase activity, elevated acetylcholine levels, reduced oxidative stress, and upregulated BDNF/TrkB signaling, among other effects [118].

Aranarochana et al. [120] demonstrated that *Prunus domestica* has notable antioxidant capacity in DPPH and FRAP assays. Regarding cognitive health and antioxidant activity, the extract of *Prunus domestica* restored SOD activity in the hippocampus and prefrontal cortex, attenuated memory deficits, and restored cell proliferation in the hippocampus of mice treated with D-galactose [120]. Exposure to *Ilex paraguariensis* prevented the reduction in astrocyte viability upon lipopolysaccharide treatment, reduced ROS and nitrite levels, and increased the expression of antioxidant genes such as *Nrf2*, *SOD2*, and *GPx* [121]. In an intranasal MPTP-induced Parkinson’s disease model in rats, pretreatment with *Eugenia uniflora* prevented the increase in ROS levels and the inhibition of the Na^+^/K^+^ ATPase enzyme in the olfactory bulb and substantia nigra, as well as lipid peroxidation in the olfactory bulb [122]. Using the same intranasal model in female rats, treatment with *Eugenia uniflora* for 14 days demonstrated neuroprotective effects by reversing MPTP-induced depression-like behaviors, increasing grooming, and reducing immobility time [123]. At the molecular level, the extract prevented the increase in α-synuclein in the hippocampus and markers such as p-53 and Bax, restoring Bcl-2 levels [123].

The benefits of plants rich in CGAs have also been explored across various models relevant to diabetes research and metabolic health (Table 2). Maiztegui et al. [6] demonstrated that *Ilex paraguariensis* extract and chlorogenic acid have antidiabetic properties, as shown by in vitro assays with isolated pancreatic islets. The same study showed that dietary supplementation with *Ilex paraguariensis* in rats improved glucose tolerance and enhanced glucose-stimulated insulin secretion in pancreatic islets, with an anti-inflammatory effect characterized by a reduction in TNF-α and PAI-1 mRNA expression [6]. Regarding the control of postprandial hyperglycemia, *Coffea arabica* extract exhibited high α-glucosidase inhibitory activity, as reported by Mitiku et al. [124]. The water extract of the Sumatra variety exhibited the highest inhibitory potency, with all tested extracts demonstrating greater inhibition of intestinal sucrase than of maltase and glucoamylase [124]. Accordingly, a CGA-rich commercial decaffeinated green coffee preparation ameliorated high-fat diet-induced glucose tolerance in mice without affecting body weight. The same study showed an improvement in brain mitochondrial energy metabolism that seems to be associated with genes involved in glucose metabolism, insulin signaling, oxidative phosphorylation, and lipid metabolism [125], highlighting the potential of green coffee in type 2 diabetes and, consequently, Alzheimer’s disease [126].

In a study investigating bone tissue homeostasis, both the aqueous extract of *Ilex paraguariensis* and the isolated chlorogenic acid increased cell viability in pre-osteoblast cells (MC3T3-E1), while only the total extract increased viability in osteocyte bone cells (MLO-Y4) [127]. In a multiple sclerosis study in mice, prophylactic treatment with *Ilex paraguariensis* reduced demyelination and infiltration of immune cells, such as T and B cells, into the central nervous system and increased the regulatory T cell population in the spleen and peripheral lymph nodes [128]. Santos et al. [129] provided an overview of the CGAs of *Ilex paraguariensis*, reporting that they can inhibit pathogenic bacteria and stimulate beneficial bacteria, such as *Bifidobacterium* and *Lactobacillus.* Moreover, Olate-Briones et al. [130] showed that *Ilex paraguariensis* reduced proinflammatory cytokines, M2 macrophage infiltration in signaling and peripheral lymph nodes, and modulated the gut microbiota in a mouse model of induced acute colitis. Using a mouse model of colitis, Liu et al. [131] observed that tea water extracts of *Camellia sinensis* attenuated weight loss, increased thigh junction protein expression, and reduced pro-inflammatory cytokine levels in the colon and liver by inhibiting the TLR4/NF-*κ*B/NLRP3 signaling pathway.

In a mouse model of gouty arthritis, treatment with *Coffea arabica*, chlorogenic acid, and neochlorogenic acid modulated both pain and inflammatory parameters. These treatments significantly reduced hypernociception, neutrophil migration, and the concentration of pro-inflammatory cytokines, including IL-1β, IL-6, and TNF-α, in periarticular tissues [132]. In addition to these inflammatory findings, *Coffea arabica* and *Coffea canephora* modulated antiatherogenic parameters in a study with rats. The extracts downregulated aortic endothelial P-selectin immunoexpression and blocked leukocyte accumulation, whereas *Coffea canephora* increased serum apolipoprotein A-1 levels [133].

The biological potential of these plant extracts extends to anticancer activity. For instance, pulps from different varieties of *Vitis vinifera* exhibited antioxidant and anticancer activities by reducing the viability of pancreatic (Capan-2) and breast (MDA-MB-231) cancer cells [134]. Similarly, *Vaccinium myrtillus* powder reduced the viability of HSC-3 cancer cells, and a concentration of 25 mg/mL inhibited the proliferation and migration of this cell line, as well as IHGK and HMK cells [135]. *Prunus avium* extract exhibited anti-mutagenic potential in 7,12-dimethylbenz(a)anthracene-induced hepatocarcinogenesis by reducing the levels of alpha-fetoprotein and carcinoembryonic antigen, as well as total serum oxidative stress, and significantly lowered levels of alanine aminotransferase, aspartate aminotransferase, and lactate dehydrogenase [136]. Regarding gene expression, there was an increase in the *CHEK1*, *CHEK2*, and *P21/CDKN1α* genes, which are linked to the inhibition of cell proliferation, and a reduction in the *ATM* gene, which is related to cell repair [136].

**Table 2:** Main biological effects investigated for plant extracts rich in chlorogenic acids.

Plant Extract	Biological Effects	References
*Camellia sinensis*	Anti-inflammatory; gut microbiota modulation	[131]
*Coffea arabica*	Anti-inflammatory; pain modulation; neuroprotection; antiatherogenic effect; antihyperglycemic effect	[118,119,124,132,133]
*Coffea canephora* (Robusta)	Antiatherogenic effect; improvement of glucose tolerance and brain mitochondrial energy metabolism	[125,133]
*Cuscutae semen*	Attenuation of depression-like behaviors; antioxidant effect; anti-inflammatory effect; gut microbiota modulation	[61]
*Eugenia uniflora*	Antioxidant effect; anti-apoptotic effect; neuroprotective effect; antidepressive-like effect	[122,123]
*Ilex paraguariensis*	Bone tissue homeostasis; anti-inflammatory effect; reduced demyelination; immunomodulatory effect; antidiabetic effect; gut microbiota modulation	[6,121,127,128,129,130]
*Malus domestica* (polyphenol-rich fraction)	Neuroprotective; gut microbiota modulation; attenuation of depression-like behaviors and cognitive impairments	[78]
*Petasites japonicus*	Attenuation of depression-like behaviors; anti-inflammatory effect; antioxidant effect; gut microbiota modulation	[62]
*Prunus avium*	Anti-mutagenic effect; antioxidant effect; hepatoprotective effect; regulation of DNA damage checkpoint pathways	[136]
*Prunus domestica*	Antioxidant effect; neuroprotective effect; attenuation of memory deficits; restored cell proliferation in the hippocampus	[120]
*Vaccinium myrtillus*	Anticancer effect	[135]
*Vitis vinifera*	Antioxidant effect; anticancer effect	[134]

Although the plant extracts analyzed contain various bioactive molecules, the attribution of the observed biological effects primarily to CGAs is supported by a convergence of experimental and structural evidence in the literature. Comparative studies reveal that isolated CGAs mimic the therapeutic outcomes obtained with crude extracts [6,132,137]. Furthermore, the synergistic effect resulting from secondary interactions with other phytoconstituents cannot be ruled out. For instance, Xu et al. highlighted the synergic interaction among CGA and caffeine on lipid metabolism in mice [138]. However, CGAs’ central role is reinforced by their modulation of canonical signaling pathways, which have been extensively described at the cellular level (see Section 3). This cause-and-effect relationship is further strengthened by computational modeling (in silico) data, which demonstrate CGAs’ ability to interact directly, stably, and thermodynamically favorably with key amino acid residues at strategic regulatory targets [98,139].

## 5. Clinical Studies

Despite robust preclinical evidence on the beneficial effects of CGAs, only a few controlled clinical studies have evaluated the effects of CGAs supplementation on human health. A modulatory action on metabolism and positive effects on cognition and the cardiovascular system by CGAs have been supported in clinical studies, although with limitations [9,140,141,142,143]. First, most research has utilized coffee extract or a mix of CGAs combined with other compounds. Second, some clinical trials evaluated a small cohort of participants over a short administration period, and many studies reported only moderate beneficial effects. Furthermore, in several cases, there were confounding factors and other aspects of the experimental design that limited the interpretation of results. In this session, we mainly discuss the effects of CGAs and CGAs-containing supplements on cognitive processes.

It has been reported that the major CGAs compounds in coffee are differentially absorbed and metabolized in humans, with large interindividual variations [27]. A study that assessed the impact of dose on the bioavailability of coffee CGAs suggested feruloylquinic acids and sulfated caffeoylquinic acid lactones as potential biomarkers in plasma and urine in humans [26]. A recent systematic review and meta-analysis conducted by Johal et al. [144] suggested that chronic coffee consumption is necessary to obtain cognitive benefits; however, the authors discussed that the number of studies analyzed was small and that a meta-analysis of randomized clinical trials was unable to confirm the benefits of coffee on cognition. The review also noted that most clinical studies have investigated the association between coffee intake and cognition; however, there is still a lack of evidence exploring the specific effects of coffee CGAs on cognition [144]. In addition, caffeine can be a confounding factor in studies with caffeinated beverages, such as coffee and mate, due to its stimulant effect and possible neuroprotective action attributed mainly to its modulatory effect on the adenosinergic system.

A pilot study with eight elderly men and women complaining of subjective memory loss showed that 330 mg of CGAs for 6 months induced a significant improvement of attentional, executive, and memory functions evaluated by the Cogstate and CNS Vital Signs test batteries [145]. In a study with a small sample of regular coffee drinkers, the participants were subjected to a double-blind acute crossover design, receiving decaffeinated green coffee, pure chlorogenic acids, or a placebo, with cognitive and mood assessments. The results varied according to the parameter evaluated, but CGAs treatment was found to improve subjective reports of tiredness, headaches, and jitteriness compared to the placebo, and these improvements were similar to those observed with decaffeinated green blend coffee [146]. In contrast, decaffeinated green coffee improved sustained attention and decision time compared to a placebo, whereas pure CGAs had no significant effect. Another randomized, placebo-controlled, double-blind study compared the effects of regular coffee, decaffeinated coffee, and placebo on measures of cognition and mood and found that some behavioral activities of coffee are beyond its caffeine content [147]. A double-blind study with 300 healthy young adult women compared the cognitive effects of Arabica and Robusta coffee and observed different results depending on the task [148]. The authors suggested that the increase in memory and efficiency of the attentional system in the *Coffea arabica* group might be due to the presence of CGAs, which are found in higher amounts in this variety in the present study. However, other studies have shown that the concentration of chlorogenic acids depends more on roasting intensity than on coffee variety [1,10,11,17], as discussed in Section 1.1.

A randomized, double-blind, placebo-controlled trial evaluated the effect of a 16-week intake of a CGAs-added beverage or placebo in middle-aged and elderly individuals and found that CGAs improved some cognitive functions, including attention and motor speed, while increasing the blood levels of apolipoprotein A1 and transthyretin, the reduced levels of which are markers of early-stage cognitive decline [149]. Ochiai et al. [150] carried out a randomized controlled crossover trial with 34 patients with mild cognitive impairment, who received an active beverage containing CGAs or a placebo twice daily for 12 weeks. The authors found that participants had a significantly reduced number of errors on the Trail Making Test, an attention task, after CGAs ingestion, but no other significant differences were observed in the examined cognitive function [150]. In contrast, a randomized, double-blind, placebo-controlled crossover study with acute administration of low and moderate doses of coffeeberry extract (standardized to 40% chlorogenic acid), or 75 mg caffeine (positive control) found that coffeeberry extract had no beneficial effect on mood, mental and physical energy levels on cognition and mood; on the contrary, acute administration of low dose increased mental fatigue during the performance of cognitively demanding tasks and decreased accuracy on a task of sustained attention, compared to placebo [151]. The authors noted that previous positive results in the literature were observed with higher doses or with extracts co-supplemented with other phenolics or administered in isolation. In another study, performance, mood, post-exercise inflammation, and oxidative stress were evaluated in cyclists who ingested 2 weeks of a high-chlorogenic-acid coffee. Total mood disturbance scores were lower with CGAs coffee, and higher levels of the ferric-reducing ability of plasma were observed after exercise compared to placebo; however, coffee did not reduce the post-exercise increased levels of inflammation or oxidative stress markers (IL-6 and hydroxyoctadecadienoic acids) [152].

Despite several preclinical and clinical studies suggesting a beneficial cognitive and neuroprotective effect of coffee, the real benefit on neurodegenerative diseases remains debatable. A recent prospective cohort study with up to 43 years of follow-up suggests that coffee and tea with higher caffeinated coffee intake, but not decaffeinated coffee, were significantly associated with a lower risk of dementia [153]. However, the amount of CGAs was not determined in the sample. In fact, the role of CGAs in coffee studies is difficult to determine because several researchers did not report the levels of CGAs and because many other compounds with neuroprotective effects are usually present in coffee preparations [154]. Table 3 summarizes the clinical studies evaluating CGAs and coffee preparations’ effects on cognitive function.

## 6. Translation Limitations and Future Directions

Chlorogenic acids are a diverse group of phenolic compounds derived from plants, present in different sources of the human diet. Preclinical studies underscore their diverse pharmacological capabilities, targeting crucial molecular pathways like Nrf2 and NF-*κ*B to deliver strong antioxidant, anti-inflammatory, neuroprotective, and antidiabetic benefits. Within the gut–brain axis, CGAs exhibit prebiotic-like effects by enhancing intestinal barrier stability and influencing gut microbiota composition. However, translating these findings into human clinical trials is challenging due to factors such as individual metabolic differences, confounding dietary factors, and differences in treatment dosages. Although smaller and acute trials have shown notable improvements in attention, motor speed, and subjective fatigue markers, larger studies are necessary to distinguish the specific therapeutic effects of isolated CGAs from those of whole-plant extracts.

An important limitation should be noted regarding the interpretation of studies that use plant extracts rich in CGAs rather than the isolated compound. These extracts frequently contain, in addition to CGAs, a wide variety of other bioactive compounds, including other polyphenols, alkaloids, terpenoids, and other secondary metabolites of the source plant, which may contribute, individually or synergistically, to the observed biological effects. Thus, it is not always possible to confidently attribute a specific therapeutic effect exclusively to the CGAs present in a complex plant extract, and this limitation should be considered in the critical interpretation of the findings discussed throughout this review.

Several factors limit the translation of the therapeutic potential of dietary supplements containing CGAs and related polyphenols into clear clinical guidelines. Firstly, a major challenge in clinical trials, including the extensive Cocoa Supplement and Multivitamin Outcomes Study (COSMOS Trial) [155], is that primary composite endpoints for global outcomes, such as total cardiovascular events or overall cognitive performance, often do not reach statistical significance. Instead, the clinical advantages tend to be selective, showing mainly as reductions in cardiovascular mortality, decreases in specific systemic inflammatory markers such as high-sensitivity C-reactive protein, or localized enhancements in episodic memory among those with initially poor dietary quality. Secondly, the presence of confounding bioactive compounds in whole-food matrix extracts, such as caffeine or various flavanols, complicates the direct attribution of clinical benefits to isolated CGAs. Lastly, there is significant interindividual variability in the systemic bioavailability of these compounds, including CGAs, which is largely dependent on host-specific intestinal microflora-mediated metabolism, which is a major confounding factor in clinical dietary supplements, influencing bioavailability, bioactive metabolite distribution, and target engagement. This issue is further limited by relatively short median trial follow-up periods, which may not capture long-term structural changes observed in several nutritional studies over extended observation periods [156,157].

Future clinical research should prioritize precision nutrition strategies that categorize subjects by initial dietary patterns, metabolic characteristics, and distinct enterotypes to enhance potential therapeutic outcomes. To establish clear causal relationships, long-term randomized controlled trials using standardized chlorogenic acid formulations free of interference from other matrix components are necessary. Future studies should conduct detailed in-person neuropsychological evaluations and monitor tissue-specific biomarkers to detect subtle structural or cognitive alterations accurately. Finally, investigating the interaction between CGAs and other metabolic treatments or probiotic strains could improve intestinal absorption and optimize overall therapeutic effects.

## Figures and Tables

**Table 1:** CGAs and CGA-rich extracts modulate critical parameters of the gut–brain axis.

Parameter	Markers	References
Cognitive dysfunction	Improved cognitive impairment, depression- and anxiety-like behaviors	[60,61,62,74,75,77,78,83,84]
Inflammation	↓ IL-6, ↓ TNF, ↓ IL-1β, ↑ IL-10, ↓ NF-*κ*B, ↓ TLR4, ↓ NLRP3, ↓ GFAP, ↓ Iba-1, ↓ COX-2	[60,61,62,74,77,83,84]
Oxidative stress	↑ Nrf2, ↑ HO-1, ↓ Keap1, ↑ SOD, ↑ CAT, ↑ GSH, ↑ GPx, ↓ MDA	[60,61,62]
Gut barrier integrity	↑ ZO-1, ↑ Occludin, and ↑ Claudin-1	[60,62,74]
Gut microbiome	↑ Community abundance and diversity, ↑ F/B ratio, ↑ SCFAs production	[60,61,62,74,75,77,78,83,84]
Neurotransmission	↑ 5-HT, ↓ 5-HIAA, ↓ Glu, ↓ GABA, ↑ DA, AChE modulation	[60,62,75,83]
Synaptic function	↑ SNAP25, ↑ PSD-95, ↑ SYP, ↑ BDNF	[62,77,84]

5-HIAA: 5-hydroxyindoleacetic acid; 5-HT: serotonin; AChE: acetylcholinesterase; BDNF: brain-derived neurotrophic factor; CAT: catalase; COX-2: cyclooxygenase-2; DA: dopamine; F/B: *Firmicutes*/*Bacteroidetes*; GABA: gamma-aminobutyric acid; GFAP: glial fibrillary acidic protein; Glu: glutamate; GPx: glutathione peroxidase; GSH: glutathione; Iba-1: ionized calcium-binding adaptor molecule 1; IL-1β: interleukin 1β; IL-10: interleukin 10; IL-6: interleukin 6; MDA: malondialdehyde; NF-*κ*B: nuclear factor kappa B; NLRP3: NLR family pyrin domain containing 3; Nrf2: nuclear factor erythroid 2-related factor 2; PSD-95: postsynaptic density protein 95; SCFAs: short-chain fatty acids; SNAP25: synaptosomal-associated protein 25; SOD: superoxide dismutase; SYP: synaptophysin; TLR4: toll-like receptor 4; TNF: tumor necrosis factor; ZO-1: zonula occludens-1; ↑: increased; ↓: decreased.

**Table 3:** Clinical studies evaluating CGAs and CGAs-rich coffee preparation effects on cognitive function.

Treatment	Study-Design	Sample	Main Results	Reference
Daily intake of 100 mL beverage containing 330 mg of CGAs for 6 months	Single arm study	Healthy elderly men and women with subjective memory loss (N = 8)	Significant improvement was observed in the One Back Test of the Cogstate, the Shifting Attention Test, and Finger Tapping Test as well as in the composite memory, verbal memory, complex attention, cognitive flexibility, executive function, and motor speed domains of the CNS Vital Signs test battery.	[145]
Acute dose of decaffeinated green blend coffee (6 g) or CGAs beverage (540 mg)	Cross-over, placebo-controlled, double blind, randomized, single center, clinical trial	Adults aged 50+ years, light to moderate coffee drinkers (N = 58)	No difference among groups regarding the primary outcome (cognitive function measures). Decaffeinated green blend coffee improved sustained attention and decision time. CGAs improved subjective reports of tiredness, headaches, and jitteriness.	[146]
Regular coffee (220 mL containing 100 mg of caffeine) or decaffeinated coffee (220 mL containing ~5 mg of caffeine)	Randomized, placebo-controlled, double-blind, counterbalanced-crossover study	Older (61–80 years, N = 30) and young (20–34 years, N = 29) adults	Regular coffee decreased reaction time and increased alertness. Decaffeinated coffee increased alertness. Higher jitteriness was observed in young females and older males after regular coffee ingestion.	[147]
*Coffea robusta* instant coffee (12 g in 100 mL of water with 4.6g of sugar) or *Coffee arabica* (3.02 g in 100 mL of water with 2.04 g of ground cardamon)	Randomized, placebo-controlled, double-blind study	Young adult women aged 18–26 years (N = 300)	*Coffee robusta* improved attention, general cognitive ability and memory, while *Coffee arabica* improved sleepiness, attention, general cognitive ability and memory and reaction time.	[148]
CGAs beverage (67.5% CQAs, 13.8 FQAs, and 18.6% diCQAs) daily for 16 weeks	Randomized, double-blind, placebo-controlled, parallel-group study	Healthy adults aged 50–69 years with subjective memory complaints (N = 38)	CGA improved motor speed, psychomotor speed, executive function, and shifting attention. CGA also increased the blood levels of the early-cognitive decline markers apolipoprotein A1 and transthyretin.	[149]
CGAs beverage (553.6 mg of CGAs in 100 mL of water containing 68.9% CQAs, 13.2% FQAs, and 17.9% diCQAs) twice daily for 12 weeks	Randomized, double-blind, placebo- controlled crossover trial	Adults aged 60–84 years with mild cognitive impairment (N = 28)	CGA improved attention and executive function in mild cognitive impairment individuals.	[150]
Coffee berry extract (100 or 300 mg, standardized to 40% CGA), or 75 mg caffeine (positive control)	Randomized, double-blind, placebo-controlled crossover study	Healthy adults aged 18–49 years	An amount of 300 mg coffeeberry extract had no effect on cognitive performance, while 100 mg of extract increased mental fatigue during cognitively demanding tasks, as well as decreased accuracy on a task of sustained attention.	[151]
High-CGA coffee (30 g in 300 mL of water prepared through Turkish method, daily for 2 weeks, containing 1066 mg of CQAs, 474 mg of caffeine and 1533 mg of total phenolics)	Randomized, double-blind, placebo-controlled, crossover study	Cyclists aged 19–51 years (N = 15)	High-CGA coffee decreased total mood disturbance scores, while it increased the ferric-reducing ability of plasma, but was not able to decrease the post-exercise increased levels of inflammation and oxidative stress markers (IL-6 and hydroxyoctadecadienoic acids).	[152]
Caffeinated coffee, decaffeinated coffee, and tea	Prospective cohort study	Health professionals from two cohorts with up to 43 years of follow-up (N = 131,821 individuals)	Higher caffeinated coffee intake was significantly associated with lower dementia risk and subjective cognitive decline. Higher intake of tea, but not decaffeinated coffee, showed similar outcomes.	[153]

CGAs: chlorogenic acids; CQA: caffeoylquinic acid; diCQA: dicaffeoyl quinic acid; FQA: feruloyl quinic acid.

## Data Availability

No new data were created or analyzed in this study. Data sharing is not applicable to this article.

## Abbreviations

The following abbreviations are used in this manuscript:

5-HIAA	5-hydroxyindoleacetic acid
5-HT	Serotonin
Aβ	Amyloid-β
AChE	Acetylcholinesterase
AKT	Protein Kinase B
AMPK	AMP-activated protein kinase
ATM	Ataxia-telangiectasia mutated gene
ARE	Antioxidant Response Element
Bax	Bcl-2-associated X protein
Bcl-2	B-cell lymphoma 2
BDNF	Brain-derived neurotrophic factor
CAT	Catalase
CDKN1A	Cyclin-Dependent Kinase Inhibitor 1A
CHEK1/2	Checkpoint kinase 1 and 2
CGA	Chlorogenic acid
CGAs	Chlorogenic acids
CQA	Caffeoylquinic acid
Cmax	Maximum plasma concentration or plasma peak concentration
COX-2	Cyclooxygenase-2
CREB	cAMP response element-binding protein
diCQA	Dicaffeoyl quinic acid
DPPH	2,2-diphenyl-1-picrylhydrazyl
Erk1/2	Extracellular signal-regulated kinases 1 and 2
F/B	*Firmicutes*/*Bacteroidetes*
FQA	Feruloyl quinic acid
FRAP	Ferric-Reducing Antioxidant Power
GABA	Gamma-aminobutyric acid
GCGR	Glucagon Receptor
GFAP	glial fibrillary acidic protein
Glu	Glutamate
GLUT4	Glucose Transporter type 4
GPx	Glutathione Peroxidase
GR	Glutathione Reductase
GSH	Glutathione
HMGCR	3-hydroxy-3-methylglutaryl-coenzyme A reductase
HO-1	heme oxygenase-1
Iba-1	ionized calcium-binding adaptor molecule 1
IL-1β	Interleukin 1 beta
IL-6	Interleukin 6
KEAP1	Kelch-like ECH-associated protein 1
LC3-II/I	Microtubule-associated protein 1 light chain 3 II/I
LDHA	Lactate dehydrogenase A
MAPK	Mitogen-activated protein kinase
MDA	Malondialdehyde
MPTP	1-methyl-4-phenyl-1,2,3,6-tetrahydropyridine
mTOR	Mechanistic target of rapamycin
Neat1	Nuclear paraspeckle assembly transcript 1
NF-*κ*B	Nuclear factor kappa B
NLRP3	NLR family pyrin domain containing 3
NPC1L1	Niemann-Pick C1-like 1 protein
Nrf2	Nuclear factor erythroid 2-related factor 2
PAI-1	Plasminogen Activator Inhibitor-1
PI3K	Phosphoinositide 3 kinase
PPARα	Peroxisome proliferator-activated receptor alpha
PSD-95	Postsynaptic density protein 95
PXR	Pregnane X receptor
ROS	Reactive Oxygen Species
SCFAs	Short-chain fatty acids
SIRT1	Sirtuin 1
SNAP25	Synaptosomal-associated protein 25
SOD	Superoxide dismutase
SREBP-2	Sterol regulatory element-binding protein 2
STING	Stimulator of interferon genes
SYP	Synaptophysin
TLR4	Toll-like receptor 4
Tmax	Time to maximum plasma concentration
TNF-α	Tumor necrosis factor alpha
ZO-1	Zonula occludens 1
